# Electromagnetic Mode Management in Transparent DMD Electrodes for High Angular Color Stability in White OLEDs

**DOI:** 10.1021/acsphotonics.4c01956

**Published:** 2025-04-10

**Authors:** Claudia Triolo, Antonella Lorusso, Sofia Masi, Fabrizio Mariano, Antonio Della Torre, Gianluca Accorsi, Valentina Arima, Stefano De Leo, Rosaria Rinaldi, Salvatore Patané, Marco Mazzeo

**Affiliations:** † Department of Civil, Energy, Environmental and Materials Engineering (DICEAM), 18976 Mediterranean University, Via Zehender, Loc. Feo di Vito, Reggio Calabria 89122, Italy; ‡ Department of Mathematics and Physics “Ennio De Giorgi”, University of Salento, Via per Arnesano, Lecce 73100, Italy; § CNR NANOTEC-Institute of Nanotechnology, Via Monteroni, Lecce 73100, Italy; ∥ Institute of Advanced Materials (INAM), Universidad Jaume I, Av. Sos Baynat s/n, Castelló 12071, Spain; ⊥ CNR IMM-Institute for Microelectronics and Microsystems, Via Monteroni, Lecce 73100, Italy; # Department of Applied Mathematics, Campinas State University, São Paulo 13083-859, Brazil; ∇ Department of Mathematical and Computer Sciences, Physical Sciences and Earth Sciences, University of Messina, Messina 98166, Italy

**Keywords:** transparent electrodes, dielectric/metal/dielectric structures, transparent white OLED, variable angle spectroscopic ellipsometry

## Abstract

The understanding and management of the optical behavior of dielectric/metal/dielectric (DMD)-based electrodes are crucial for the design of fully transparent OLEDs. Specifically, the chromatic stability with the viewing angle of white OLED emission remains an important issue due to the angle dependence of internal reflection at the organic/electrode interface as the wavelength varies. The purpose of the present work is to provide a complete analysis of the optical behavior of DMD structures by Variable Angle Spectroscopic Ellipsometry in order to optimize the transmittance of DMD-based transparent white OLEDs over a wide viewing angle. The analysis of the optical modes contributing to power dissipation reveals that the reduction of plasmonic and waveguided modes is related to the antireflection properties of the DMD electrode. This results in a simultaneous significant improvement in the absolute value of the transmittance across the full visible spectral range and in the color stability of white OLED emission over a wide viewing cone of 120°, thus paving the way for a new generation of transparent white lighting sources.

## Graphical abstract

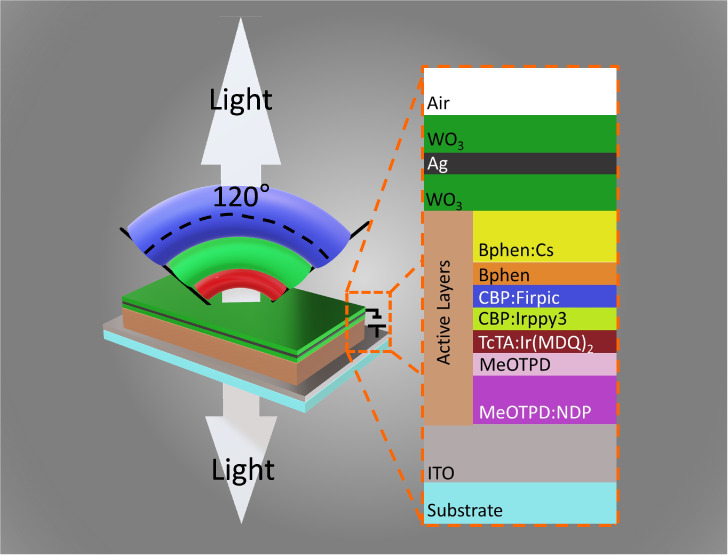

## Introduction

The integration of photonic structures in optoelectronic devices represents an ever-evolving research field.^1–4^ In these devices, the photonic structures allow for manipulating light propagation, acting on its reflection, absorption, transmission, and polarization state.^5–9^ In particular, the next generation of transparent organic light-emitting diodes (OLEDs) will be used for several applications, such as see-through displays, augmented reality/virtual reality head-mounted displays, and smart windows in architecture, as well as fully transparent computer displays, smart glasses, and the integration of fully transparent OLEDs in automotive windshields and military applications.^7,8,10–15^

The realization of high-performance fully transparent OLEDs remains a significant challenge due to the trade-off between the transparency and conductivity of the top and bottom electrodes. For this purpose, transparent conductive oxides and very thin metal films have been utilized as transparent and semitransparent electrodes.^4,9,16,17^ In particular, indium–tin-oxide (ITO) is widely used in optoelectronic devices due to its low sheet resistance and high transmittance in the visible region (over 90%).^16,18^ Nevertheless, ITO is rarely used as a cathode in top-emission OLEDs, since the high deposition temperature can damage the underlying organic layers.^19^ On the other hand, very thin semitransparent metal films can promote an island-like morphology, which is responsible for reduced film conductivity and transmittance due to the scattering of incident light.^20^ Moreover, the reflectivity of the metal layer induces microcavity effects that seriously limit the efficiency of transparent white OLEDs, giving rise to an angular dependence of the color emission.^21–26^ In the last years, layered structures of metal-dielectric thin film materials (such as metal/oxides or metal/high gap semiconductors) have been proposed as transparent electrodes.^27–29^ The transparency of the structure is attributed to two alternative mechanisms: (i) the suppression of surface plasmon polariton (SPP) coupling at the metal/dielectric interface;^30^ (ii) the dielectric function mismatch between the dielectric and metal layers.^31^ A unifying model that describes the optical characteristics of the metal/dielectric thin films used as electrodes is still missing, even for dielectric-metal-dielectric (DMD) trilayer structures.^6,11,32–36^ So far, a large part of the reported studies is limited to the normal incidence of light, without any correlation among reflectance, transmittance, and waveguide modes.^37^ A full understanding of the electromagnetic wave propagation at the layers’ interface could lead to designing the electrode to achieve the best optical transparency, resulting in transparent OLEDs with very high angular chromatic stability, which is still limited by the wavelength responsivity of the DMD electrodes.

In this work, we demonstrate how to realize transparent electrodes based on DMD architecture for white OLED applications, effectively managing the electromagnetic transversal electric (TE or s) and transversal magnetic (TM or p) modes. This results in an optimization of the transmittance of the DMD electrode with high angular color stability. We analyze the optical behavior of a WO_3_/Ag/WO_3_ sequence using Variable Angle Spectroscopic Ellipsometry (VASE) to understand the relationship between the transmittance of the structure and the TM and TE modes of the reflected light at different dielectric thicknesses. Proper matching of the thickness and dielectric functions of the layers included in the DMD structure, along with the illumination conditions (incidence angle and wavelength of light), ensures a minimization of total reflection of light and a maximization of the transmittance of the transparent electrode. This optimization occurs under critical conditions of incidence angle and wavelength, which depend on the dielectric thickness, but positive effects on the reflectance and transmittance through a DMD structure are also observable at normal incidence. This behavior applies to both light directions, namely light entering the device and light exiting the device through the DMD electrode.

Such experimental results have been explained by the application of a phasor-Fresnel coefficient-based model to a multilayer configuration. Our findings show that such DMD electrodes are able to maximize the transmittance in a very large visible spectral range (450**–**680 nm). Moreover, the spectral emission is fully stabilized under a large angular viewing in a cone of 120° aperture, with the color rendering index (CRI) remaining fixed at a value of 80 within this aperture cone and the color Commission Internationale de l’Eclairage coordinates (CIE x, y) varying by just 0.01, thus demonstrating how DMD technology can be useful for lighting applications.

## Experimental Section

From VASE analysis on DMD structures, the refractive index and effective thickness of both the top and bottom WO_3_ layers were evaluated in the visible range using the Cauchy model with the following equation $n\left(\lambda \right)=A+\frac{B}{\lambda^{2}}+\frac{C}{\lambda^{4}}$ . Meanwhile, the same parameters for the Ag layer were estimated using the Drude-Lorentz oscillators model (see Figure S1 for details).

A complete Cryo-FIB/SEM system (AMBER-Tescan) has been used to perform a measurement of the trilayer section deposited on a glass substrate (see Figure S2). A platform composed of an electron beam and an ion beam that can be used separately for observation and ion milling within a single instrument. Thanks to the presence of a FEG source and 4 different electron detectors, the minimum resolution for SEM in high vacuum at room temperature and in cryogenic conditions is 0.9 nm @ 15 kV, whereas the FIB resolution in high vacuum at room temperature and in cryogenic conditions is <2.5 nm @ 30 keV. In particular, the deposited film was first removed using the ion beam, and then SEM analysis was performed to observe the cross-section of the deposited material.

The doped *p-i-n* stack transparent white OLED in top and bottom emission has been produced using a thermal evaporation technique in a LESKER Super-Spectros deposition tool. The device was sequentially deposited in a high vacuum chamber (10^–6^ mbar) at a rate of 0.5–1 Å/s on ITO-coated glass substrates. Before deposition, the substrates were sonicated in acetone for 10 min and then in isopropyl alcohol, dried with nitrogen, and finally cleaned using UV ozone treatment prior to use. The transparent white OLED architecture, with a total organic stack of 120 nm, consists of: glass/110 nm of ITO as the bottom transparent electrode/40 nm of MeOTPD:NDP (namely N,N,N’,N’-tetrakis­(4-methoxyphenyl)­benzidine doped with 2-(7-dicyanomethylene-1,3,4,5,6,8,9,10-octafluoro-7H-pyrene-2-ylidene)-malononitrile), used as the hole injection layer (HIL)/10 nm of MeOTPD used as the hole transport layer (HTL)/27 nm of emissive layer (EML)/10 nm of Bphen (4,7-diphenyl-1,10-phenanthroline) used as the electron transport layer (ETL)/40 nm of Bphen:Cs used as the electron injection layer/DMD as the top transparent electrode. Specifically, the EML includes FIrPiC (bis­[2-(4,6-difluorophenyl)­pyridinato-C2,N]­(picolinato)­iridium) as the blue emitter, Ir­(ppy)_3_ (tris­(2-phenylpyridine)­iridium­(III)) as the green emitter, and Ir­(MDQ)­2­(acac) (bis­(2-methyldibenzo­[f,h]­quinoxaline) (acetylacetonate)­iridium­(III)) as the red emitter. The Ir­(MDQ)_2_(acac) is mixed with TCTA (tris­(4-carbazoyl-9-ylphenyl)­amine), which is used as the electron blocking layer (EBL), to form 7 nm of TCTA:Ir­(MDQ)_2_(acac). Meanwhile, FIrPiC and Ir­(ppy)_3_ are mixed with CBP (4,4’-bis­(N-carbazolyl)-1,1’-biphenyl), which is used as the hole blocking layer (HBL), to form two layers of 10 nm thickness each: CBP: FIrPiC and CBP: Ir­(ppy)_3_.

## Results and Discussion

### Optical Characterization of the DMD Structures

The optical response of the DMD electrodes has been studied using the VASE technique (Figure 1a). Broadly, ellipsometric analysis allows for measuring the change in the polarization state between the incident and reflected light as the angle of incidence varies. The polarization change is described by the ellipsometric angles Ψ and Δ, which are related to each other by the equation: ρ = tan­(Ψ)*e* ^iΔ^, where Δ = *δ*_*p*_ – *δ*_*s*_ is the phase difference between the reflected p and s modes, and tan­(Ψ) = |*r*_*p*_|/|*r*_*s*_| depends on the ratio of the complex reflection coefficients of the p and s modes (*r*_*p*_,*r*_*s*_), respectively. These variables are functions of the optical dispersion and the material thickness under study.

**Figure 1:**
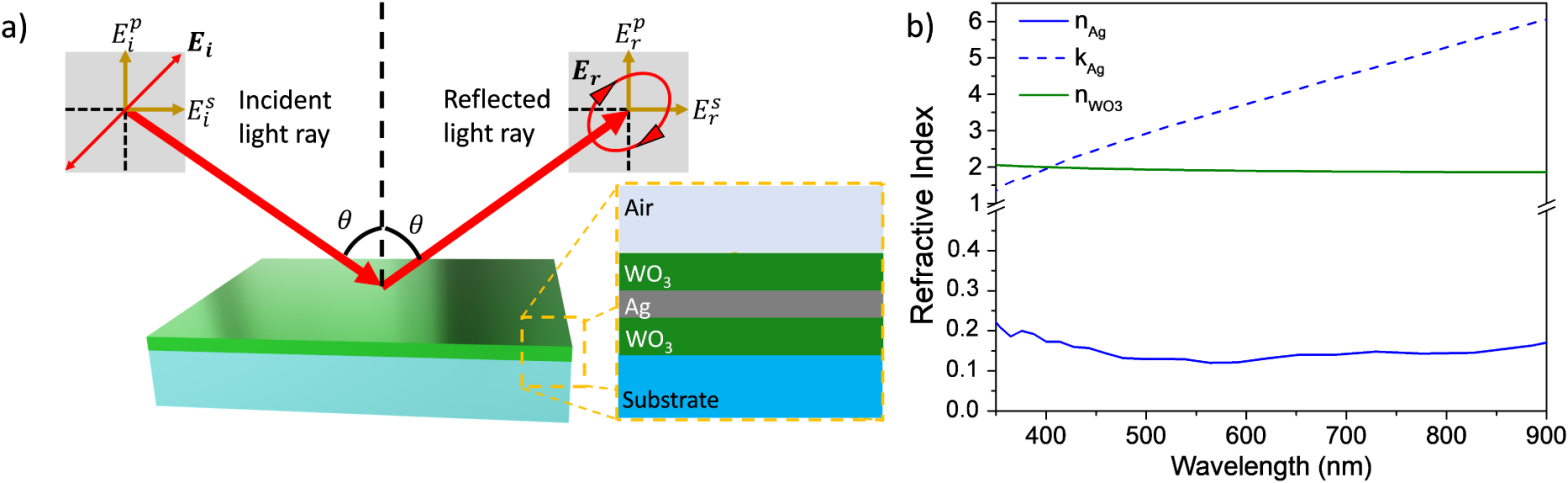
(a) Scheme of VASE setup: a light linearly polarized at 45° with respect to the incidence plane impinges the upper layer of the DMD structure with an angle of incidence θ and then reflected back. The change in the electric field polarization is determined through the ellipsometric angles Δ and Ψ. (b) Wavelength-dependence of the real (continuous blue line) and imaginary (dashed blue line) part of the complex refractive index of Ag (*ñ*_Ag_ = *n*_Ag_ + *ik*_Ag_) layer and refractive index (real, *n*_Wo3_) of WO_3_ layer as calculated from Δ and Ψ.

A series of DMD structures were built by considering an Ag thickness *d*_*Ag*_ of 15 nm, which ensures quite uniform coverage,^18^ while the WO_3_ dielectric thickness was changed from 15 to 70 nm. Figure 1b shows the optical dispersion curves for the silver layer and the real refractive index of the WO_3_ thin film. The optical dispersion of the metal layer (*ñ*_Ag_ = *n*_Ag_ + *ik*_Ag_) evidences a real refractive index *n*_Ag_ of about 0.15 in the orange-NIR region (550–900 nm) that increases up to 0.25 at shorter wavelengths. The absorption coefficient *k*_Ag_ has an almost linear dependence on the wavelength. A first analysis of the optical dispersion curves for the DMD structure suggests that, at short wavelengths, the contribution of WO_3_ is predominant; thus, the reflectance mainly depends on the optical dispersion of the dielectric. As the wavelength increases, the contribution of *k*_Ag_ linearly rises in the NIR region until it prevails over the dielectric contribution.

The polarization state of the reflected light can be represented on the Poincaré sphere, which is a convenient method to visualize the polarization state of light through the normalized Stokes vector, whose components are related to the ellipsometric angles Ψ and Δ by the following relations:

1 $$S_{1}=-\cos\left(2\Psi \right)$$

2 $$S_{2}=\sin\left(2\Psi \right)\cdot \cos\left(\Delta \right)$$

3 $$S_{3}=-\sin\left(2\Psi \right)\cdot \sin\left(\Delta \right)$$

These components identify a point on a unitary Poincaré sphere that indicates the polarization state of the light, as shown in Figure 2a. The points on the equatorial plane indicate a linear polarization state of light; the point at (1,0,0) indicates a pure p mode, while the opposite point (−1,0,0) indicates a pure s mode. The upper and lower poles represent right- and left- hand circular polarization states, respectively. Any other point on the Poincaré sphere indicates an elliptical polarization state, for which the rotation sense of the circular component is determined by the location in the upper or lower hemisphere (i.e., right or left hand).^38^

**Figure 2:**
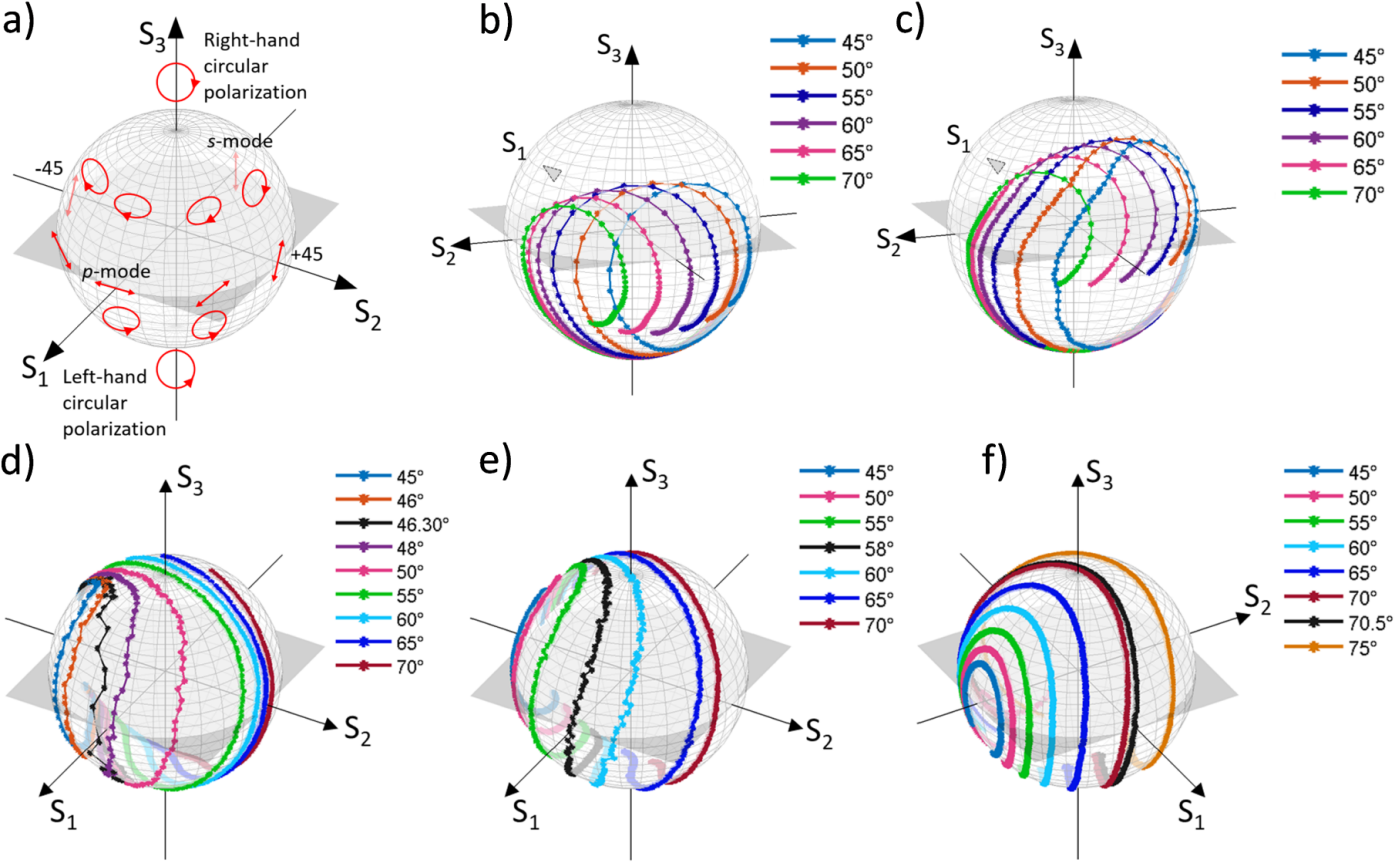
(a) Poincaré-sphere representation of the polarization state of light. Constant angle curves (CACs) as a function of the light wavelength for different dielectric thicknesses: (b) DMD15, (c) DMD30, (d) DMD40, (e) DMD55, and (f) DMD70. In panels d–f, the black line indicates the polarization state curve acquired at θ*_c_*.

Figure 2b–f shows the Poincaré sphere representations of the polarization state of reflected light from the DMDX structures investigated, where X indicates the thickness of the dielectric layer. Each colored curve on the Poincaré sphere has been acquired at a fixed angle of incidence (constant angle curves—CACs) while varying the wavelength of the excitation light.

For DMD15 and DMD30, all CACs have a spiral-like shape (Figure 2b,c) and shift along S_2_S_3_ plane as the incidence angle increases (toward S_2_ positive). At longer wavelengths, the curves tend to the (0, −1, 0) point, which corresponds to a mode −45° polarized. This is due to the contribution of the Ag layer to reflection, which dominates at longer wavelengths, thus converting the incident 45° polarized light to −45°.

Interestingly, a different optical behavior is observed for DMD40, DMD55, and DMD70 structures. In fact, the optical signals exhibit some similar features, regardless of the dielectric thickness: (*i*) with a decreasing angle of incidence of light, the curves on the Poincaré spheres tend to collapse to the point (0, −1, 0), as already observed for DMD15 and DMD30; (*ii*) a curve (the black points in Figure 2d–f) that passes through the point (1, 0, 0) does exist. These black CACs are acquired by varying the wavelength and fixing a specific value of the incidence angle of the light, named the critical angle, *θ*_*c*_. At a precise incident light wavelength (critical wavelength, *λ*_*c*_) on this curve, the reflected light is fully p-polarized, a condition that corresponds to the coordinates (1, 0, 0) on the Poincaré sphere. There, the ellipsometric parameters take the values: Ψ ≅ π/2 and *r*_*s*_(*θ*_*c*_;*λ*_*c*_) ≅ 0. The critical values (*θ*_*c*_ and *λ*_*c*_) increase proportionally to the dielectric thickness, resulting in 46.3° at 460 nm for DMD40, 58° at 550 nm for DMD55, and 70.5° at 680 nm for DMD70, respectively.

Interestingly, by fixing the incidence angle and varying the wavelength of the excitation light, each curve covers a significant portion of the surface of the Poincaré sphere, thus indicating that several polarization states may be achieved by our structures, including circular polarization. This condition occurs at λ=398 nm and θ = 55° for DMD40, λ = 477 nm and θ = 60° for DMD55, and λ = 543 nm and θ = 70° for DMD70, thus spanning the visible spectrum from blue to orange. Moreover, even unpolarized incident light could achieve a fully polarized state after reflection from the DMD structure (when properly engineered ). The above analysis demonstrates that it is possible to manipulate the polarization state of the light reflected from a DMD structure, and certain critical conditions can be selected to obtain a fully p-polarized reflected signal, resembling the Brewster angle of a simple dielectric plane but in TM mode.

As expected, the dependence of the polarization states of the reflected light on the incidence angle and wavelengths affects the reflectance and transmittance features of the DMD structures. Considering the absorbance *A*, the transmittance *T* can be written as $T=1-A-\frac{R_{p}}{2}\left(1+\tan^{-2}\Psi \right)$ , where the *R*_p_ is the reflectance of the p mode. This relationship holds for a 45° polarized incident light, as in our case, where the reflection coefficient is the arithmetic average between p and s modes, namely, $R=\frac{R_{p}+R_{s}}{2}$ . In other words, the maximum value of *T* occurs at Ψ ≅ π/2, which coincides with the critical illumination conditions. It is clear from the previous relationship that when Ψ ≅ π/2, the function tan^–2^(Ψ) has a minimum with zero and therefore transmittance remains almost flat around both the critical angle and wavelength. If *R*_p_ is also minimized, then *T* will be further maximized.

Figure 3a,b shows the reflectance and transmittance spectra acquired at normal incidence for all DMD structures produced. Interestingly, in the investigated visible spectral range, at a wavelength of 450 nm, the DMD40 structure exhibits almost zero reflectance and a maximum transmittance of 82%. Moreover, in the DMD40 electrode, the transmittance intensity remains above 75% across a wide wavelength range from 420 to 600 nm, which is typically used for white lighting applications. These results represent the best performance among those obtained from all DMD structures produced. For other dielectric thicknesses, the reflectance does not reach zero at any wavelength, likely due to a residual contribution of the s mode that, under critical conditions (see Poincaré-sphere analysis), is completely removed (Figure 3a). In fact, for the other DMD structures, the transmittance is reduced compared to DMD40, with a progressive widening and shift toward longer wavelengths of the transmittance bands as the thickness of the dielectric layer increases (Figure 3b).

**Figure 3:**
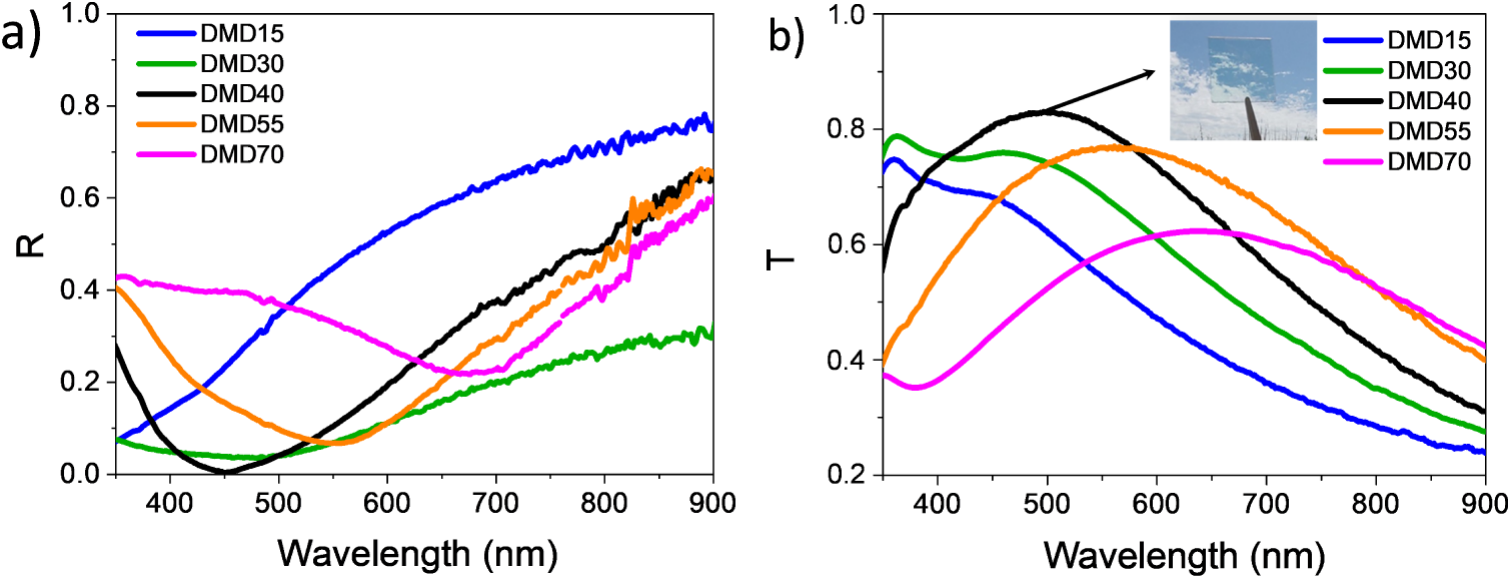
(a) Total reflectance and (b) transmittance spectra of produced DMD structures, acquired at normal incidence as a function of the incident wavelength. The inset picture shows the transparency of the DMD40 electrode.

### Theoretical Interpretation of the Optical Features of the DMD Structures

Starting from this preliminary study, the complex reflection coefficients of the p and s modes, resulting from the multiple reflections at the DMD interfaces, have been calculated by means of a phasor-Fresnel coefficient-based model for two different light propagation directions: upward direction (the light comes from the external environment) and downward direction (the light comes from the emissive layers inside the OLED and is reflected in the backward direction). In order to find analytical solutions, we consider that the top and bottom WO_3_ layers have the same thickness *d* and refractive index *n*, while the real part of the Ag refractive index has been neglected (*ñ*_Ag_ ≅ *ik*_Ag_), due to its negligible contribution, as shown in Figure 1b. Taking into account the layered structure of the DMD, we can write the Fresnel coefficients as (see further details reported in the Supporting Information):

4 $$r_{12}=-r_{23}=\frac{n\cos\theta -i\sqrt{m^{2}+n^{2}\sin^{2}\theta }}{n\cos\theta +i\sqrt{m^{2}+n^{2}\sin^{2}\theta }}$$

5 $$r_{34}=\frac{n\cos\theta -\sqrt{1-n^{2}\sin^{2}\theta }}{n\cos\theta +\sqrt{1-n^{2}\sin^{2}\theta }}$$

Here, *r*_12_ and *r*_23_ are the reflection coefficients at the interface between medium 1 and medium 2, namely WO_3_-bottom and the Ag layer, and medium 3 is the upper WO_3_, respectively; θ is the incidence angle of a ray striking the 1–2 interface from WO_3_-bottom. Moreover, *r*_34_ is real in the angular range $0\leq \theta \leq \arcsin\frac{1}{n}$ , namely between 0° and 36°. Under these assumptions, the zero-reflectance condition leads to the evaluation of metal (d*) and dielectric (*d*) thickness as a function of the dielectric constant of materials, wavelength, and incidence angles (see the Supporting Information for more details), allowing for the engineering of the DMD electrode.

This model has been utilized to evaluate the reflectivity of the light emitted by the chromophores at the organic/DMD electrode interface and to establish the antireflection conditions. In Figure 4, the s mode reflectance as a function of the dielectric thickness of the DMD structures and the incidence angle has been plotted at three fixed wavelengths (470 nm, 550 and 630 nm), for both downward (left side of Figure 4) and upward (right side of Figure 4) directions. The wavelengths have been chosen to correspond to the emission wavelengths of the active layers inside the OLED. As a general trend, the s mode reflection exhibits a minimum (<1%, in both light incidence directions) that shifts toward thicker values of the dielectric layer and greater angles of incidence as the wavelength of incident light increases. The evident difference between the maps obtained in the two different light propagation directions is due to the position of the minima, which are shifted toward lower incidence angles in the maps obtained for the downward direction compared to the maps obtained for the opposite direction. This is attributed to the total internal reflection that involves light emitted from the active layers inside the OLED devices. Regardless of the incoming direction of light, the antireflection conditions are validated in both cases. Hence, the VASE measurements are an optimal tool to obtain information about the reflections that occur inside the device, which are not experimentally accessible with conventional optical tools, such as ellipsometry.

**Figure 4:**
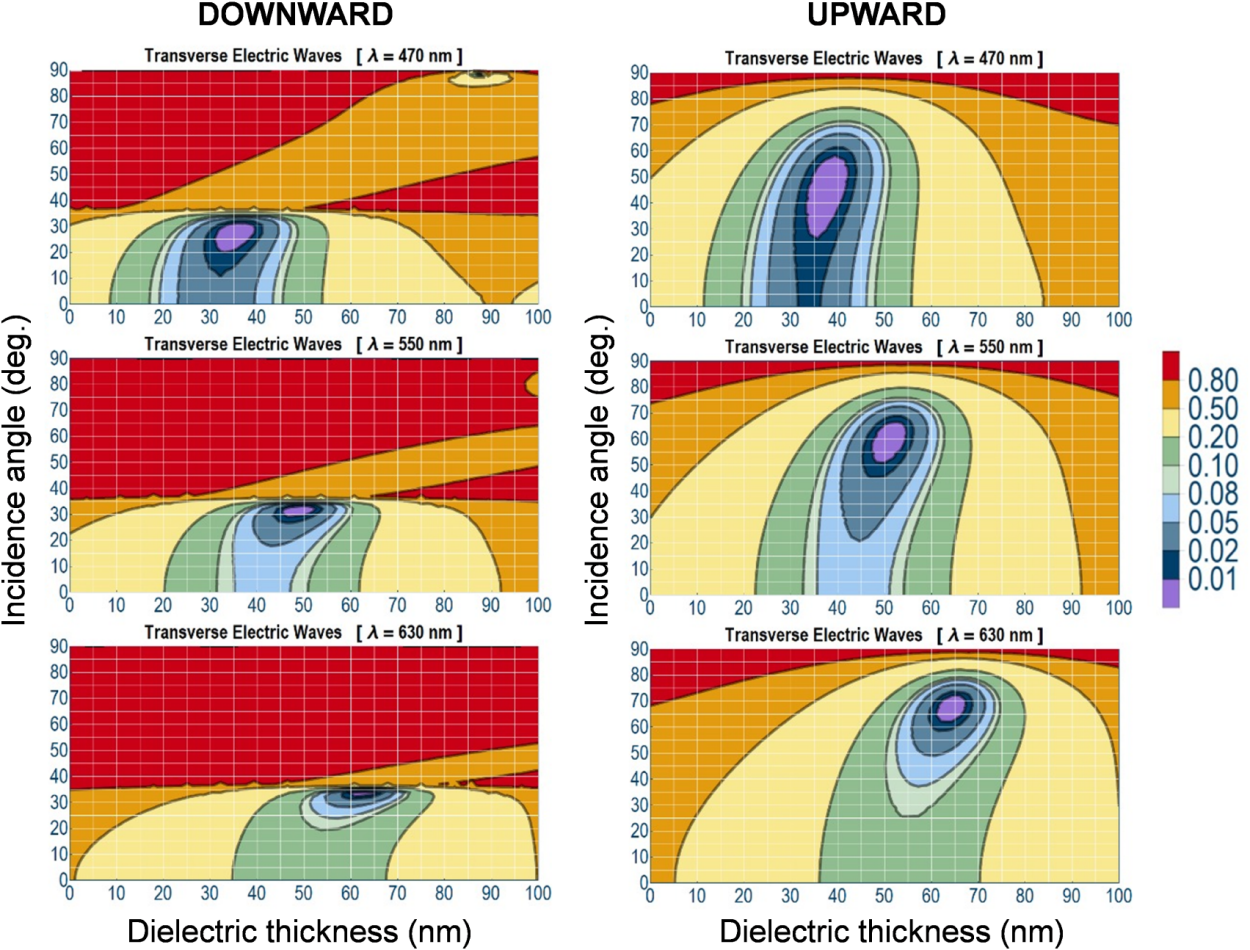
Reflection of s mode as a function of the dielectric thickness and the angle of the light incidence for downward (left) and upward (right) directions.

The p mode shows similar behavior (see Figure S3): in both light incidence directions, the p mode reflection also shows a minimum that shifts toward thicker values of the dielectric layer as the wavelength of incident light increases. However, there are some differences in the reflection of p and s modes: in the downward direction, the minima position of the p mode in the maps is located at lower thicknesses of the dielectric layer (ranging between 10 and 20 nm); in the upward direction, antireflection conditions are achieved at the same dielectric thickness values as the s mode, but slightly shifted toward larger angles of incidence. In both cases, the minimization of the reflection is more effective for the s mode (as already observed in the Poincaré-sphere representation), while a large part of the p-polarized light is lost since it is reflected in the backward direction, inside the OLED, contributing to the waveguided modes.

Considering an effective refractive index of 1.7 for the full organic stack, the out-coupled light escapes from the OLED device at an angle of approximately 60° with respect to the normal at the surface (i.e., an aperture cone of 120°), which corresponds roughly to an internal angle lower than 36°. We underline that the dielectric thickness of 40 nm is a good compromise, allowing for low reflectance at different wavelengths, thus keeping the spectral shape of transmittance highly stable as the internal incidence angle increases.

### Realization and Investigation of a Transparent White OLED Using DMD40 as the Upper Transparent Electrode

We have finally fabricated a *p-i-n* transparent white OLED, whose architecture is shown in Figure 5a, while further details are reported in the Experimental Section. White electroluminescence (EL) has been obtained by the overlap of the emission signals from FIrPic, emitting at 467 nm, Ir­(ppy)_3_ emitting at 514 nm, and Ir­(MDQ)_2_(acac) emitting at 616 nm. Owing to singlet-to-triplet overlap states, these fluorescent compounds display a large Stokes shift, allowing light emission in the visible range and absorption in the ultraviolet, thus not being detrimental to the transmittance in the visible range. Images of the switched-off and switched-on device are also reported in Figure 5a.

**Figure 5:**
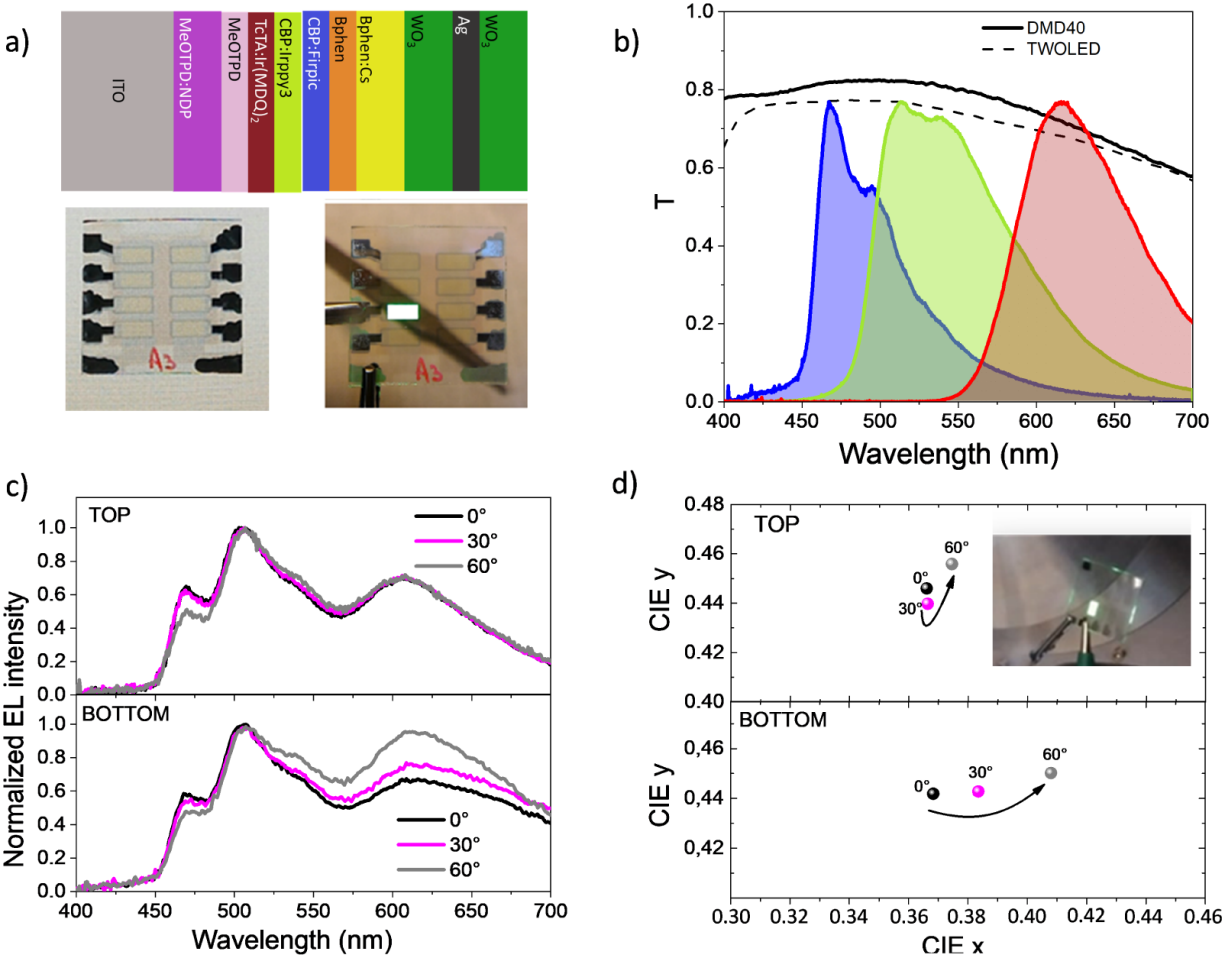
(a) Architecture of the transparent white OLED with the pictures of the switched-off and switched-on device. (b) Transmittance curves of the DMD40 electrode (continuous line) and transparent white OLED (dashed line). The emission spectra of the emitting RGB materials used are also reported. (c) Viewing angle-dependent spectra of top and bottom emission at 0°, 30°, and 60° to the direction normal to the substrate. (d) CIE coordinates of the out-coupled light from the top and bottom transparent white OLED in the viewing angle range of 0–60°. The inset shows a switch-on transparent white OLED in a tilted viewing angle.

The light transmittance through the entire device is shown in Figure 5b, compared to that of DMD40 alone. The transmittance of both is characterized by a broad spectral response that covers the entire visible range, with a maximum of 78% and 82% for the transparent white OLED and DMD40, respectively. The EL signal of the transparent white OLED as a function of the wavelength is shown in Figure 5c for the light emitted through the DMD40 electrode (namely, top emission) and through the ITO electrode (namely, bottom emission). The EL spectra have been detected at three different viewing angles, namely 0°, 30°, and 60°, and normalized with respect to the maximum value of the green peak. Regardless of the viewing angles, the EL signal is characterized by three distinct peaks that correspond to the EL signal of the individual active RGB materials, as shown in Figure 5b. Moreover, when varying the viewing angles, a great spectral stability characterizes the EL signal from the DMD electrode. On the contrary, the light escaping from the bottom side, i.e., the ITO electrode, is reflected back at the Organic/ITO and ITO/Glass interfaces with an intensity strongly dependent on the angle. Since the red wavelength is less guided at the ITO/Glass interface, we expect that all these processes will produce a large variation in the transmitted spectrum toward the red region from the ITO side while being suppressed from the DMD electrode, as observed.

These optical features of the emitted light result in very small changes in the CIExy color coordinates, as reported in Figure 5d. The CIE coordinates for the top emission vary from (0.36, 0.44) at 0° to (0.37, 0.45) at 60° (Δ*x* = 0.01 and Δ*y* = 0.01). The CRI data exhibit a negligible variation around 80, indicative of good color rendering performance. Measurements of 79.98, 79.89, and 79.93 have been obtained at viewing angles of 0°, 30°, and 60°, respectively (Figure S4). On the contrary, for the bottom emission, the CIE coordinates vary from (0.37, 0.44) at 0° to (0.41, 0.45) at 60°, with coordinate changes of Δ*x* = 0.04 and Δ*y* = 0.01, showing a significantly greater variation than the top emission. The color stability with the visual angle can be maintained during the aging of the device and, in general, under different luminance conditions due to the decoupling of the optical behavior of the DMD electrode and the electrical behavior of the full OLED.

### Theoretical Interpretation of the Power Dissipation Modes

A simulation code based on the transfer matrix was used to assess the impact of the DMD architecture as the top electrode on the transparent white OLED, justifying the properties observed in the EL spectra. Figure 6 shows the power dissipation maps vs the wavevector normalized in-plane, namely defined as *u*_**||**_ = *k*_**||**_/*k*_**0**_,^39–41^ with *k*_0_ = 2π/λ. Panel (a) shows the power dissipation map for a simple DM40 structure, where the upper dielectric layer has been removed; panels (b–d) show the dissipated power maps for DMD40, DMD55, and DMD70 structures, respectively. It is possible to distinguish four regions:^39–41^ (1) *u*_**||**_ < 0.5 corresponds to the out-coupled light, (2) 0.5 ≤ *u*_**||**_ < 0.8 corresponds to the internal reflection, (3) 0.8 ≤ *u*_**||**_ < 1 is attributed to the waveguide modes, and finally, (4) *u*_**||**_ ≥ 1 refers to the coupling with SPPs. For the DM40 used as the top electrode, a large fraction of light is trapped in reflection modes, two very close and sharp waveguide modes, and finally, an intense SPP mode.

**Figure 6:**
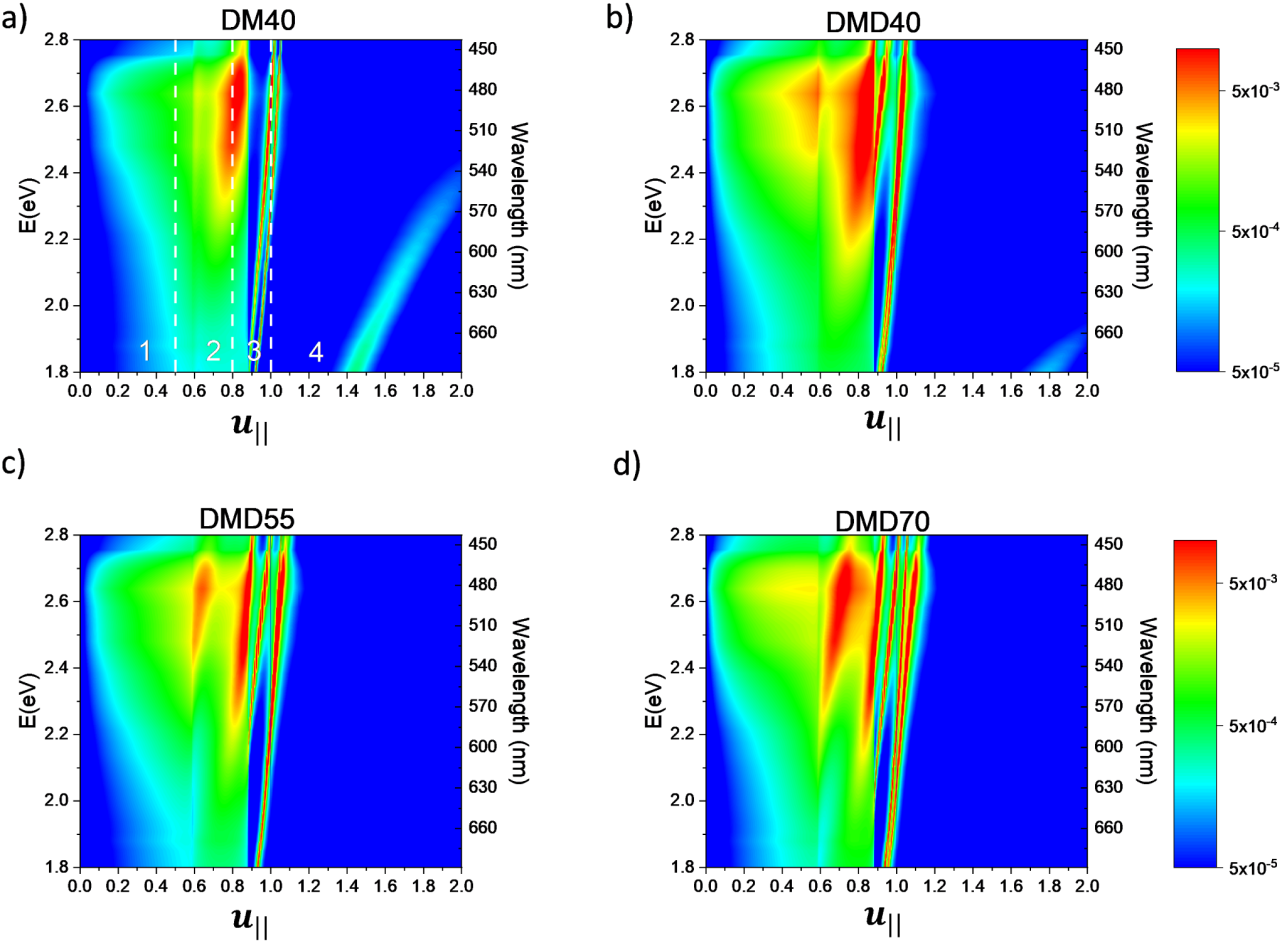
Simulation of power dissipation maps of white OLED with different types of top electrode: (a) DM40, (b) DMD40, (c) DMD55, and (d) DMD70. The numbers in panel a indicate the different power dissipation modes: (1) light emitted into air from the OLED; (2) light trapped inside the substrate; (3) waveguided modes, and (4) SPP.

In the DMD40 configuration (Figure 6b), the out-coupled light is significantly enhanced compared to DM and the other DMD electrodes, as a consequence of larger transmittance properties in a wider spectral region. Moreover, the SPP modes dramatically vanish due to the coupling of the electromagnetic wave with the top dielectric layer, thereby bypassing the coupled plasmonic oscillations.

However, due to the upper dielectric layer, an additional guided mode appears at shorter wavelengths (Figure 6b), becoming more pronounced as the dielectric thickness increases (Figure 6c,d). From these findings, we can conclude that the DMD40 electrode is capable of coupling to far-field light across a wide spectral range for white light applications.

In order to prove the potential benefit of the DMD electrode in OLEDs, we compared DM- and DMD-electrode-based devices in terms of the fraction of power dissipated into the different optical modes as a function of the dielectric thickness. Figure 7 shows that the main fraction of power is lost into the organic and dielectric layers by internal reflection and waveguided modes (indicated as WG in the panels). Moreover, SPP modes, which travel at the interface between the metal and the dielectric layers, are more effective in DM devices at longer wavelengths.

**Figure 7:**
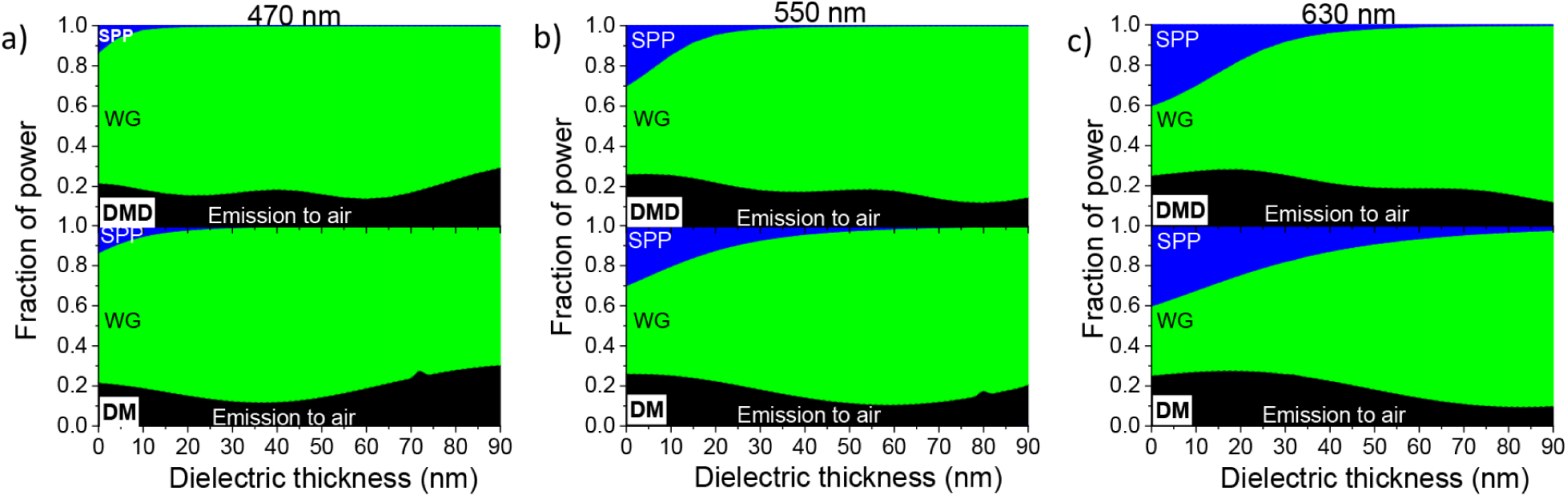
Fraction of power at the three emission wavelengths for the top-emitting transparent white OLED device indicated with the DMD label in comparison with DM electrode as a function of dielectric thickness.

Interestingly, in the DM-based devices, for the three wavelengths, the out-coupled light shows a minimum value at WO_3_ thicknesses of 40 nm, 55 nm, and 70 nm, which correspond to the maximum of the out-coupled light when the upper dielectric layer is deposited (DMD configuration). This confirms that the improvement in the out-coupled light is attributed to the antireflection properties of the electrode, which reduces the waveguide modes, boosting the direct emission to air. Moreover, the DMD with a dielectric thickness of 40 nm is the only configuration with a relative maximum in the out-coupled light for all the considered wavelengths, showing a positive impact on the possibility of realizing transparent white light-emitting devices, in which a wide range of optical frequencies are out-coupled.

## Conclusions

In conclusion, this study provides a complete analysis of the optical response of the DMD electrodes, based on WO_3_/Ag/WO_3_ layers, used in transparent white OLEDs. The data acquired by VASE in reflection mode allow for understanding the relationship between the transparency of the DMD to light incoming from outdoors and the polarization states of the reflected light. The analysis of the polarization state of the reflected light from the DMD structures as a function of the incidence angle and wavelength allows for determining the critical conditions to obtain a fully p-polarized reflected signal. Antireflection conditions are theoretically calculated by using the phasor-Fresnel coefficient-based model, in both downward and upward directions, proving that they are almost independent of the incoming direction of light. Hence, the VASE technique is an optimal tool to obtain information about the reflections that occur inside the device, which are not directly accessible.

Due to its better transparency in the visible range, the DMD40 has been used as the top electrode in a transparent white OLED based on a three-primary RGB color emission. We observe that both the plasmonic and waveguided modes are reduced owing to the antireflection properties of the DMD electrode. This behavior results in very high angular stability of the EL signal with negligible color coordinate variation within an escaping light cone of 120°, thus paving the way for a new generation of transparent white lighting sources.

## Supplementary Material



## Notes

Further details about VASE method to extract thickness and refractive index of materials, the analytical approach of the phasor-Fresnel coefficient-based model for the layered structure of the DMD; reflection of p mode as a function of the dielectric thickness and the angle of the light incidence for downward (left) and upward (right) directions (PDF)
