# Supplementary material for: Electromagnetic Mode Management in Transparent DMD Electrodes for High Angular Color Stability in White OLEDs
Source: ACS Photonics. 2025 Apr 10;12(5):2413–22. doi: 10.1021/acsphotonics.4c01956 (PMC12107611; doi:10.1021/acsphotonics.4c01956)
Supplement: Supplementary file 1 [file ph4c01956_si_001.pdf]

Supporting Information

Electromagnetic Mode Management in Transparent DMD Electrodes for High Angular Color Stability in White OLEDs.

Claudia Triolo, Antonella Lorusso*, Sofia Masi, Fabrizio Mariano, Antonio Della Torre, Gianluca Accorsi, Valentina Arima, Stefano De Leo, Rosaria Rinaldi, Salvatore Patané and Marco Mazzeo**

C. Triolo
Department of Civil, Energy, Environmental and Materials Engineering (DICEAM), Mediterranean University, Via Zehender, Loc. Feo di Vito, 89122 Reggio Calabria, Italy.
E-mail: claudia.triolo@unirc.it
orcid.org/0000-0001-9263-7786

A. Lorusso, M. Mazzeo
Department of Mathematics and Physics "Ennio De Giorgi", University of Salento, Via per Arnesano, 73100, Lecce, Italy; CNR NANOTEC-Institute of Nanotechnology, Via Monteroni, 73100 Lecce, Italy.
E-mail: antonella.lorusso@unisalento.it; marco.mazzeo@unisalento.it
orcid.org/0000-0003-4657-7334, orcid.org/0000-0001-5401-801X

S. Masi
Institute of Advanced Materials (INAM), Universidad Jaume I, Av. Sos Baynat s/n, 12071 Castelló, Spain.

F. Mariano, G. Accorsi, V. Arima
CNR NANOTEC-Institute of Nanotechnology, Via Monteroni, 73100 Lecce, Italy.

A. Della Torre
CNR IMM-Institute for Microelectronics and Microsystems, Via Monteroni, 73100 Lecce, Italy

S. De Leo
Department of Applied Mathematics, 13083-859, Campinas State University, SP, Brazil.

R. Rinaldi,
Department of Mathematics and Physics "Ennio De Giorgi", University of Salento, Via per Arnesano, 73100, Lecce, Italy; CNR IMM-Institute for Microelectronics and Microsystems, Via Monteroni, 73100 Lecce, Italy

S. Patané
Department of Mathematical and Computer Sciences, Physical Sciences and Earth Sciences, University of Messina, 98166 Messina, Italy.

VASE method to extract thickness and refractive index of materials.

In order to find the real thickness and refractive index of WO_3 and Ag, we perform ellipsometric measurements on three different structures: 1) WO_3 single layer deposited on glass, named D, 2) WO_3/Ag layers deposited on glass, named DM and 3) the WO_3/Ag/WO_3 layers deposited on Glass, named DMD. Figure S1 shows the data and fit results for nominal thickness of 30 nm, 15 nm and 30 nm for WO_3 bottom, Ag and WO_3 top layers respectively, in the three configurations. The Cauchy and Drude-Lorentz models for WO_3 and Ag layers have been used to reproduce the ψ and Δ ellipsometric data, finding excellent fits. The fitting result is a thickness of the WO_3 bottom of about 32 nm in the D configuration and n=1.85 (see also Figure 1b). These thickness and refractive index values have been fixed for fitting the DM configuration, where only the Ag thickness and refractive index have been used as free parameters in the Drude-Lorentz model. In this case, an Ag thickness of about 14 nm is found and the dispersion curves are reported in Figure 1b. Finally, in the DMD configuration, the previous values have been fixed while only the WO_3 top thickness and refractive index have been used as free parameters in the Cauchy model, finding a thickness of about 29 nm and a refractive index similar to that of the bottom layer. The same procedure has been replied for the other thicknesses.

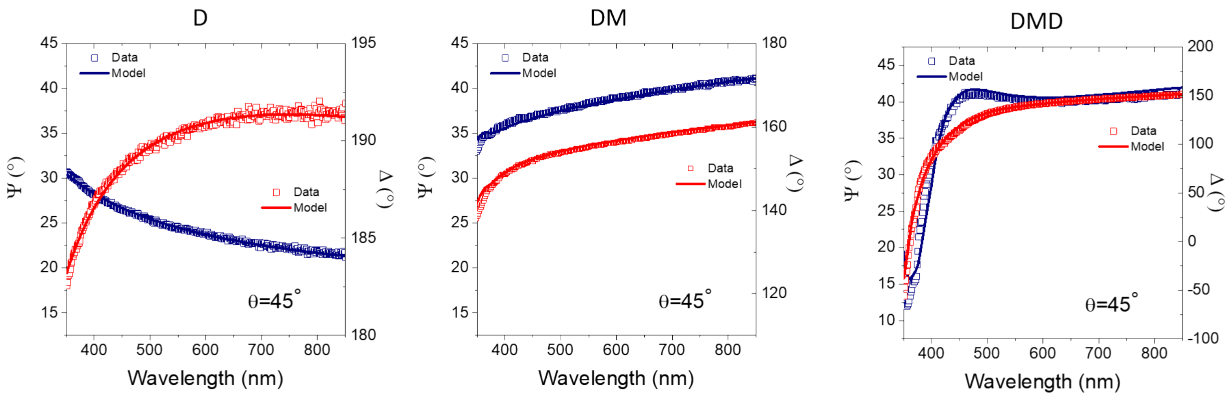

Figure. S1: Ellipsometric data and model, at incident angle of 45°, for WO_3, WO_3/Ag and WO_3/Ag/WO_3 layers deposited on Glass, named D, DM and DMD respectively.

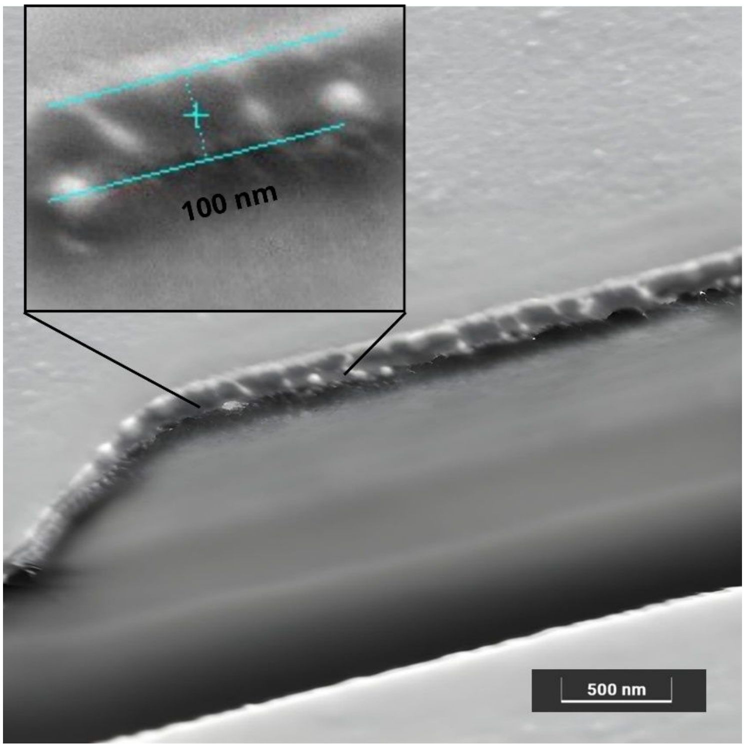

Figure. S2: Cryo-FIB/SEM image cross-section of DMD40. As we can see, the section of the deposited film is composed of layers with a total thickness of about 100 nm in agreement with the thickness achieved by ellipsometric measurements (WO_3/Ag/WO_3, 40 nm/15 nm/40 nm, respectively) and, in particular, we can observe a slight difference in contrast in the central part of the trilayers, due to the thin Ag layer (15 nm) placed between the two dielectric layers.

Theoretical model

The s-mode reflection coefficient at the interface 1-2, namely r_{12}, is opposite to that at interface 2-3, namely r_{23}, giving

$$r_{12} = -r_{23} = \frac{ncos\theta - i\sqrt{m^2+n^2sin^2\theta}}{ncos\theta + i\sqrt{m^2+n^2sin^2\theta}} = e^{-i2\psi} \quad (1)$$

where θ is the incidence angle from medium 1 (dielectric-bottom) to medium 2 (Ag-layer), n is the refractive index of the dielectric layer and m is the imaginary part of refractive index of the Ag layer, while $\psi = arctan\frac{\sqrt{m^2+n^2sin^2\theta}}{ncos\theta}$.

The reflection coefficient at the interface 3-4, namely r_{34} between medium 3 (top dielectric) and medium 4 (air) is

$$r_{34} = \frac{ncos\theta - \sqrt{1-n^2sin^2\theta}}{ncos\theta + \sqrt{1-n^2sin^2\theta}} = \begin{Bmatrix} \rho & if\ nsin\theta < 1 \\ e^{-i2\alpha} & if\ nsin\theta > 1 \end{Bmatrix} \quad (2)$$

where $\alpha = arctan\frac{\sqrt{n^2sin^2\theta-1}}{ncos\theta}$

In the case of $nsin\theta > 1$, we have evanescent transmitted waves and the light is almost totally back-reflected.

On the contrary, when $nsin\theta < 1$, corresponding to the angle range where light is able to escape outside the device, we have that the antireflection condition is given by (see ref. [32])

$$r_{12} + r_{23}e^{i2q_2d_2} + r_{34}e^{i2q_2d_2}e^{i2q_3d_3} + r_{12}r_{23}r_{34}e^{i2q_3d_3} = 0 \quad (3)$$

where $q_xd_x = \frac{4\pi}{\lambda}n_xd_xcos\theta_x$, with n_x, d_x, and θ_x are the refractive index, the thickness of the medium 2 or 3 and $\theta_x$is the refractive index into the medium 2 or 3. In the case of metal layer n_2 $= im$ is imaginary giving a negative exponential contribution, meaning absorption. In our configuration light is coming from the bottom WO_3 (medium 1), then being reflected back at the interface WO_3 bottom-Ag (medium 1-2) giving a Fresnel coefficient r_{12}, passes through the Ag layer and it is reflected back at interface Ag-WO_3 top (medium 2-3), thus contributing with the term $r_{23}e^{i2q_2d_2}$. Since q_2 is imaginary due to the refractive index of the metal layer, this exponential corresponds to an absorption coefficient given by $e^{-2|q_2|d_2}$. Then light passes through WO_3-top being reflected at interface 3-4, namely WO_3 top-air, giving a contribution r_{34} $e^{-2|q_2|d_2}e^{i2q_3d_3}$. The last three-order term came from the multiple reflection inside the layers. Therefore, in order to satisfy the antireflection conditions, we obtain from Eq.3

$$r_{12} - r_{12}e^{-2|q_2|d_2} + r_{34}e^{i2|q_3|d_3}e^{-2|q_2|d_2} - r_{34}{r_{12}}^2e^{i2|q_3|d_3} = 0$$

from which, taking in account equations 1 and 2,

$$e^{-i2\psi}(1 - e^{-2|q_2|d_2}) + \rho e^{i2|q_3|d_3}(e^{-2|q_2|d_2} - e^{-i4\psi}) = 0 \quad (4)$$

A simple algebraic manipulation leads to

$$(1 - e^{-2|q_2|d_2})^2 = \rho^2(e^{-i4\psi} - e^{-2|q_2|d_2})(e^{i4\psi} - e^{-2|q_2|d_2})$$

$$\left(1-\rho^2\right)e^{-4|q_2|d_2}+\left(1-\rho^2\right)-2(1-\rho^2cos4\psi)e^{-2|q_2|d_2}=0$$

From which we can get the absorption coefficient into the metal layer:

$$e^{-2|q_2|d_2}=\frac{1-\rho^2cos4\psi}{1-\rho^2}-\sqrt{\frac{1-\rho^2cos4\psi}{1-\rho^2}-1}=\tilde{f}$$

Moreover from eq. 4, we get

$$e^{-i2|q_3|d_3}e^{-i2\psi}\left(1-e^{-2|q_2|d_2}\right)=-\rho\left(e^{-2|q_2|d_2}-e^{-i4\psi}\right)$$

The real part being

$$\cos(2|q_3|d_3-2\psi)\left(1-\tilde{f}\right)=\rho\left(cos4\psi-\tilde{f}\right)$$

while the imaginary part is

$$\sin(2|q_3|d_3-2\psi)\left(1-\tilde{f}\right)=\rho sin4\psi$$

From which

$$|q_3|d_3=-\psi+\frac{1}{2}arctan\frac{sin4\psi}{cos4\psi-\tilde{f}}=\tilde{g}$$

From these equations, we can get the thickness of the metal and dielectric layers which satisfy the antireflection conditions as functions of the incident angle and wavelength, namely:

$$d_2=d_{Ag}=\frac{\lambda ln\,(1/\tilde{f})}{4\pi\sqrt{m^2+n^2sin^2\theta}}$$

$$d_1=d_3=d_{WO3}=\frac{\lambda\tilde{g}}{2\pi ncos\theta}$$

The reason for neglecting the real part of the Ag refractive index is due to the negligible contribution on the optical path. Indeed, the phase of the reflected light by Ag and WO_3 bottom layer is $\varphi'=\frac{4\pi}{\lambda}\cos\theta(2n_{WO3}d_{WO3}+n_{Ag}d_{Ag})$. If we ignore the real part of Ag refractive index the phase becomes $\varphi=\frac{4\pi}{\lambda}\cos\theta(2n_{WO3}d_{WO3})$. Therefore, the percentage variation of the phase is $\frac{\varphi'-\varphi}{\varphi}=\frac{n_{Ag}d_{Ag}}{2n_{WO3}d_{WO3}}\approx 0.01$, resulting in a variation of just 1%.

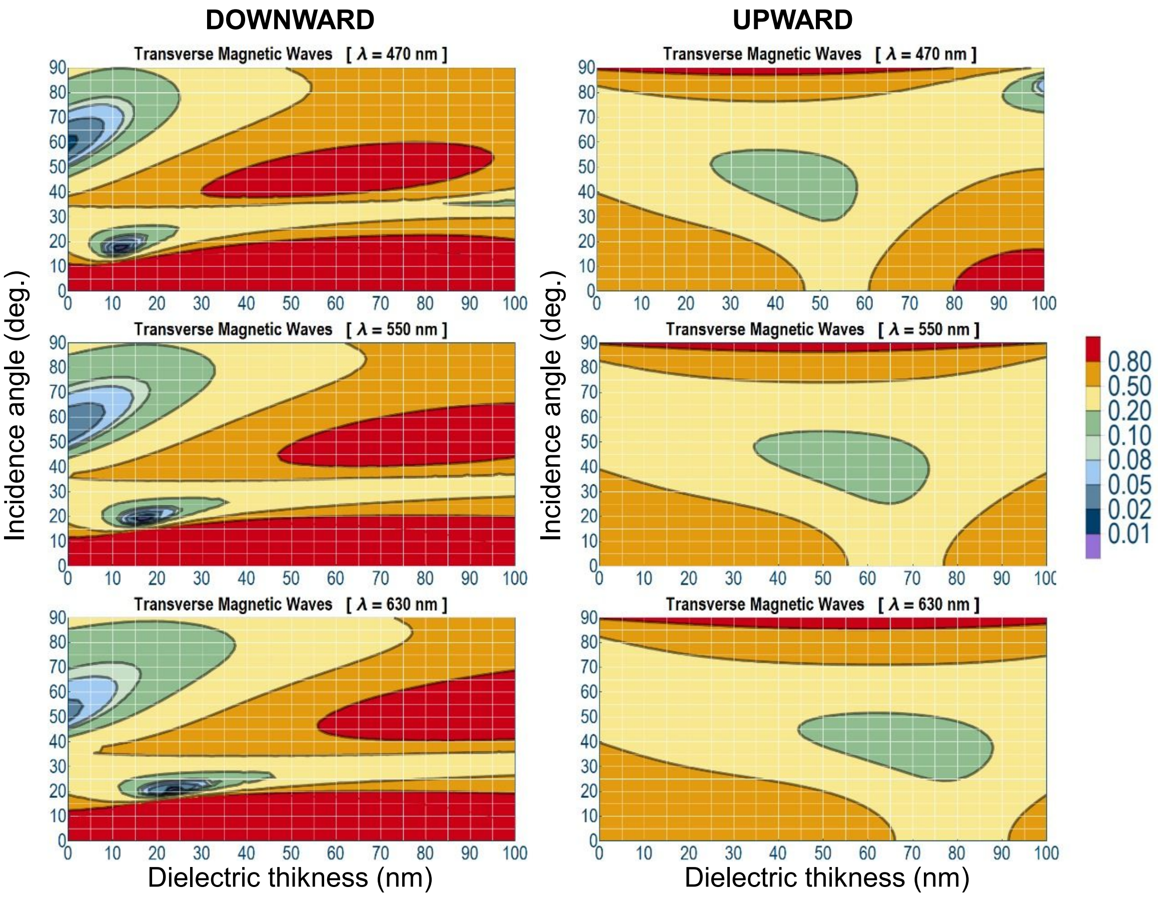

Figure S3: Reflection of p-mode as a function of the dielectric thickness and the angle of the light incidence for downward (left) and upward (right) directions.

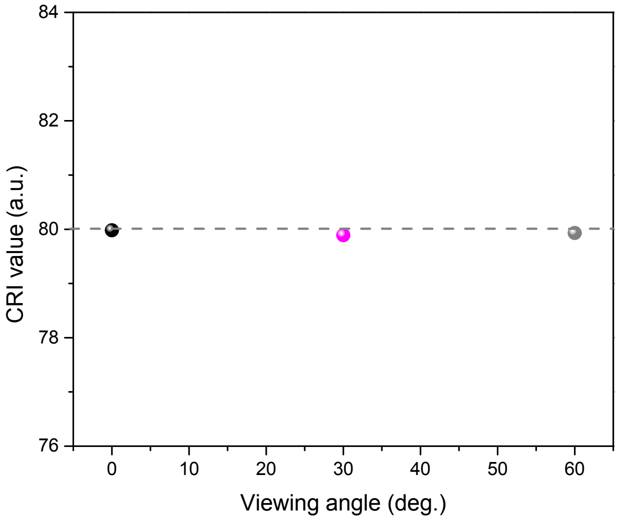

Figure S4: CRI values for DMD transparent electrode (top emission from OLED) as a function of the viewing angle (0°, 30° and 60°). The dashed lines at 80 has been added to evidence the very small variation of the CRI value of the DMD as the viewing angle varies.
